# Intellectually able adults with autism spectrum disorder show typical resting-state EEG activity

**DOI:** 10.1038/s41598-022-22597-z

**Published:** 2022-11-08

**Authors:** Qianliang Li, Ricarda F. Weiland, Ivana Konvalinka, Huibert D. Mansvelder, Tobias S. Andersen, Dirk J. A. Smit, Sander Begeer, Klaus Linkenkaer-Hansen

**Affiliations:** 1 Section for Cognitive Systems, Department of Applied Mathematics and Computer Science (DTU Compute), Technical University of Denmark, 2800 Kongens Lyngby, Denmark; 2 Department of Integrative Neurophysiology, Center for Neurogenomics and Cognitive Research (CNCR), Amsterdam Neuroscience, Vrije Universiteit Amsterdam, 1081 HV Amsterdam, The Netherlands; 3 Faculty of Behavioural and Movement Sciences, Department of Clinical- Neuro- and Developmental Psychology, Vrije Universiteit Amsterdam, Amsterdam Public Health Research Institute, 1081 HV Amsterdam, The Netherlands; 4 Amsterdam Neuroscience, Department of Psychiatry, Amsterdam UMC, University of Amsterdam, 1012 WX Amsterdam, The Netherlands

**Keywords:** Neuroscience, Biomarkers, Autism spectrum disorders, Machine learning

## Abstract

There is broad interest in discovering quantifiable physiological biomarkers for psychiatric disorders to aid diagnostic assessment. However, finding biomarkers for autism spectrum disorder (ASD) has proven particularly difficult, partly due to high heterogeneity. Here, we recorded five minutes eyes-closed rest electroencephalography (EEG) from 186 adults (51% with ASD and 49% without ASD) and investigated the potential of EEG biomarkers to classify ASD using three conventional machine learning models with two-layer cross-validation. Comprehensive characterization of spectral, temporal and spatial dimensions of source-modelled EEG resulted in 3443 biomarkers per recording. We found no significant group-mean or group-variance differences for any of the EEG features. Interestingly, we obtained validation accuracies above 80%; however, the best machine learning model merely distinguished ASD from the non-autistic comparison group with a mean balanced test accuracy of 56% on the entirely unseen test set. The large drop in model performance between validation and testing, stress the importance of rigorous model evaluation, and further highlights the high heterogeneity in ASD. Overall, the lack of significant differences and weak classification indicates that, at the group level, intellectually able adults with ASD show remarkably typical resting-state EEG.

## Introduction

Autism spectrum disorder (ASD) is defined by persistent differences in social interactions, atypical sensory reactivity, and restricted and repetitive behavior^1^. High heterogeneity exists among individuals receiving the diagnosis, since the criteria allow a broad spectrum of symptoms, and the neural mechanisms underlying ASD remain unclear. To elucidate the neurobiological mechanisms behind ASD, many studies have used neuroimaging^2–4^. Discovery of biomarkers for ASD has the potential to support diagnosis and might disentangle the heterogeneity^5^. Recently, there has been a growing interest in discovering resting-state electroencephalographic (EEG) biomarkers for various neuropsychiatric conditions, as EEG has good clinical practicality, due to being non-invasive, portable, widely available, and low cost.

Many different resting-state EEG features have been investigated in regards to ASD, with spectral power being the most commonly used feature. Decreased theta power^6^, alpha power^7–10^ and gamma power have been observed in ASD^11,12^. However, increased alpha power^13^ and gamma power have also been reported in ASD^14^. Other spectral features, e.g., peak alpha frequency^15^, theta/beta ratio^16^ and asymmetry^17^ have also been associated with ASD. Besides spectral features, abnormal functional connectivity^18^, microstates^19^, and measurements of criticality^20,21^ have also been found in ASD. The role of each individual feature and their implications on ASD are outside the scope of this paper (for reviews, see^2,3,22^).

In addition to identifying group mean differences of resting-state EEG features in ASD, many recent studies also investigated the potential of classifying ASD with predictive machine learning models. The advantages of predictive modelling is that it is optimized for predicting new subjects or future outcomes, which aligns with the goal of diagnostic/prognostic tools^23^. Different models and features have been employed, e.g., discriminant function analysis using spectral power have yielded 90% accuracy^16^ and using coherence 87% balanced accuracy was obtained^24^. K-nearest neighbor using alpha power resulted in 96.4% accuracy, and support vector machines (SVM) have also been utilized with alpha power and peak alpha frequency and achieved 93% accuracy^15^. SVM have also been employed on recurrence quantification analysis features, which resulted in 92.9% accuracy^25^, and on 20 features consisting of some descriptive statistics (e.g. mean and variance), spectral power, Hjorth parameters and entropy features, which resulted in 93.8% accuracy^26^. Besides conventional machine learning models, artificial neural networks have also been used for classification of ASD and obtained 94.8%^27^, 97.2%^28^ and 100%^29–31^ accuracy. However, not all studies have reported such promising results, one study using discriminant function analysis with spectral power obtained 64.5% balanced accuracy^32^, and another study using SVM with spectral power obtained 68% accuracy^33^. A recently published paper using SVM on spectral power and coherence in over 400 subjects also reported around 57% accuracy^34^. The differences in classification performances may relate to the heterogeneity and complexity of ASD^5^; however, other sources of uncertainty about the exact classification performance include low sample sizes, imbalanced datasets, and lack of an unseen test dataset. Specifically, some of the machine learning studies did not use an independent test set or cross-validation to split the data^10,25,33^ or only used a single layer of cross-validation for training, hyperparameter tuning and testing^26,27,29,31,35^. Both of these situations are problematic due to the evaluated classification performances being likely to be overestimated due to overfitting (reviewed in^36–39^). Additionally, only five of the resting-state EEG studies classifying ASD investigated more than 100 subjects^15,16,24,32,34^, thus currently there is no clear consensus about the physiological resting-state EEG correlates of ASD.

**Figure 1:**
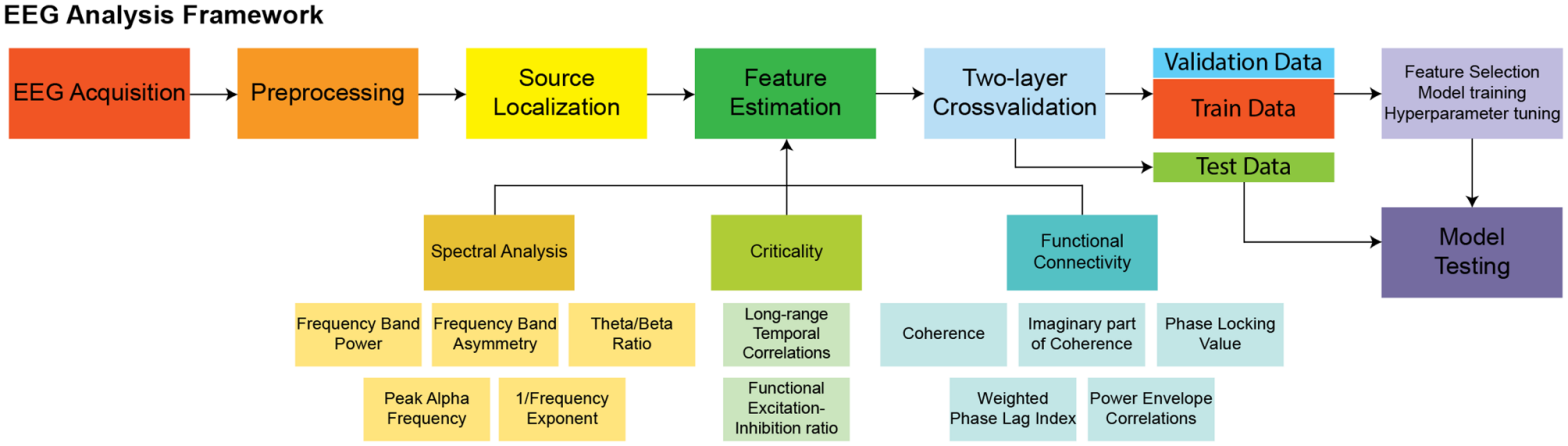
Overview of the EEG analysis framework. The EEG data was preprocessed and source modelled, followed by estimation of commonly used EEG biomarkers. Repeated 10-by-10 two-layer cross-validation was performed to split the data. Feature selection, model training and hyperparameter tuning were performed on the inner fold training and validation set, while the generalization performance was evaluated on the entirely unseen test set. Logistic regression, random forest, and support vector machine were employed to classify ASD.

To disentangle the potential role of resting-state EEG biomarkers for characterization of ASD, the present study estimated many of the commonly used resting-state EEG features that have shown promising results. Specifically, we source-modelled the EEG time series and computed spectral features (power, asymmetry, theta/beta ratio, peak alpha frequency and 1/f exponent), measures of criticality (long-range temporal correlations [DFA exponent] and functional excitation inhibition ratio [fEI]) and functional connectivity (coherence [Coh], imaginary part of coherence [Imcoh], phase locking value [PLV], weighted phase lag index [wPLI] and power envelope correlations [PEC]). A relatively large sample size of 186 participants were recruited and feature selection methods and machine learning models were applied to evaluate the biomarker potential of the selected EEG features in distinguishing between individuals with and without ASD. To not have our results be dependent on one particular machine learning model, we trained variations of three commonly used classifiers, namely support vector machine (SVM), logistic regression with L1 and L2 norm and random forest. Additionally, to obtain a robust evaluation of the model performances, we employed repeated two-layer cross-validation, in order to train the models on a training set, estimate hyperparameters on a validation set and finally evaluate the generalization performance on an unseen separate test set (see Fig. 1 for overall analysis framework). To our knowledge, no studies aiming to predict ASD using resting-state EEG with machine learning have employed such a comprehensive EEG biomarker set, and stringent repeated cross-validation scheme for evaluating their models.

## Results

### Multiple EEG features exhibited correlation with age

For each EEG feature type, we computed how many features were significantly correlated with age after false discovery rate (FDR) correction (Pearson’s correlations, $p<$ 0.05) and observed that spectral power, theta/beta ratio, 1/f exponents and PEC had greater than chance-level correlations with age. Table 1 shows the percentage of all features within each feature type that were significantly correlated with age.

**Table 1:** Percentage of significant correlations with age.

Power	Theta/beta	Asymmetry	Peak Alpha Freq
**27.2**	**100**	0.0	1.4

1/f	Coh	Imcoh	PLV
**73.5**	0.0	0.0	0.0

wPLI	PEC	DFA	fEI
0.0	**11.1**	1.2	0.3

Numbers in bold indicate more than 5% of the features were significantly correlated with age after FDR correction.

**Figure 2:**
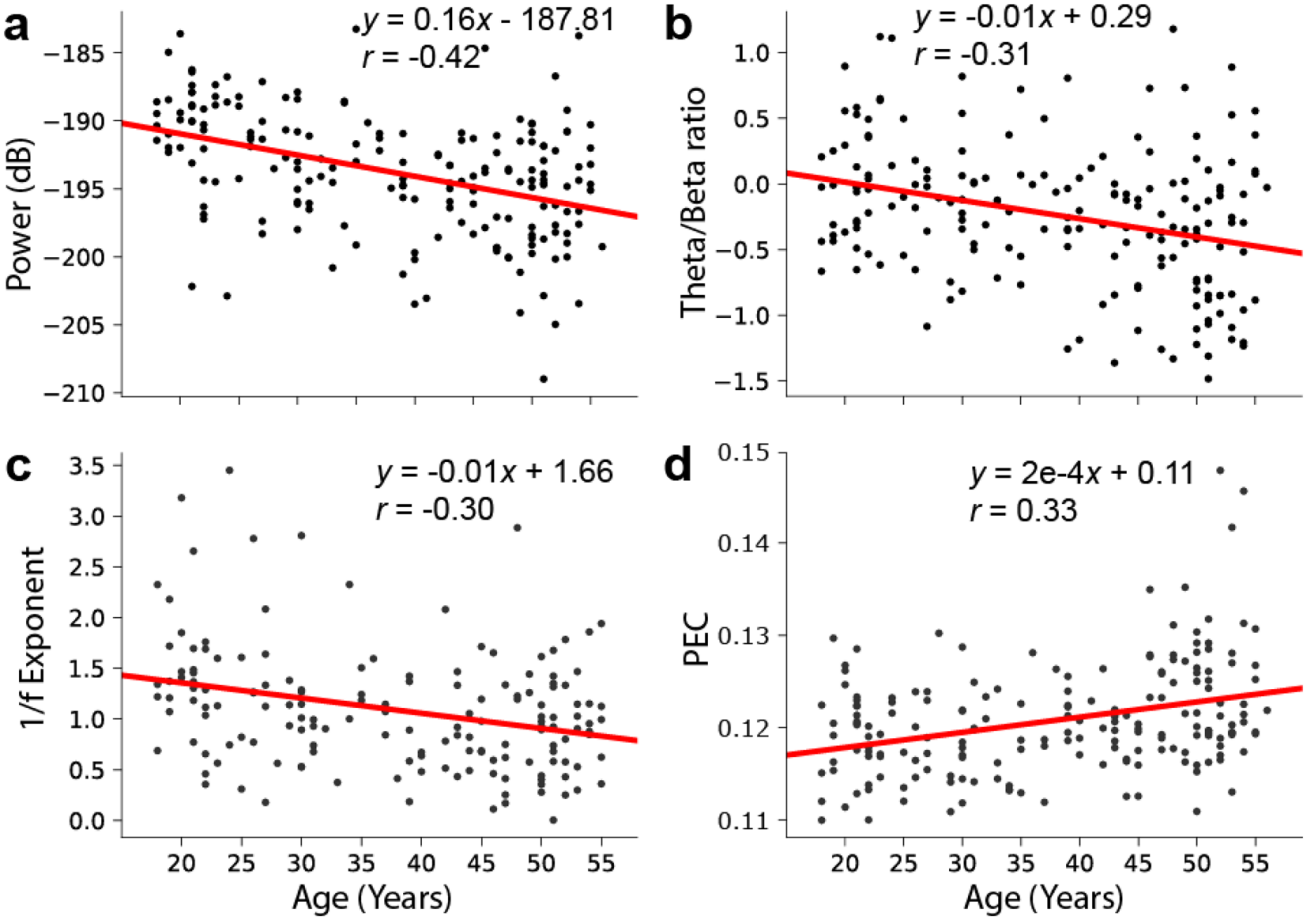
Several EEG features exhibit age dependence. Scatter plots illustrating correlations between age and (**a**) Absolute Power, (**b**) Theta/Beta Ratio, (**c**) 1/f exponent, and (**d**) PEC of source-modelled signals.

Further investigation revealed that the correlations between power, PEC and age were frequency-band dependent. Specifically, absolute delta, theta and alpha power, relative beta and gamma power, and delta PEC exhibited high correlations with age. To take into account the difference in age between the ASD and non-autistic comparison groups, we removed the linear trend for 1/f exponents, theta/beta ratio, and power and PEC in the above-mentioned frequency bands. Figure 2 show examples of the strongest correlations with age for each of the highlighted feature types prior to age-effect correction.

**Figure 3:**
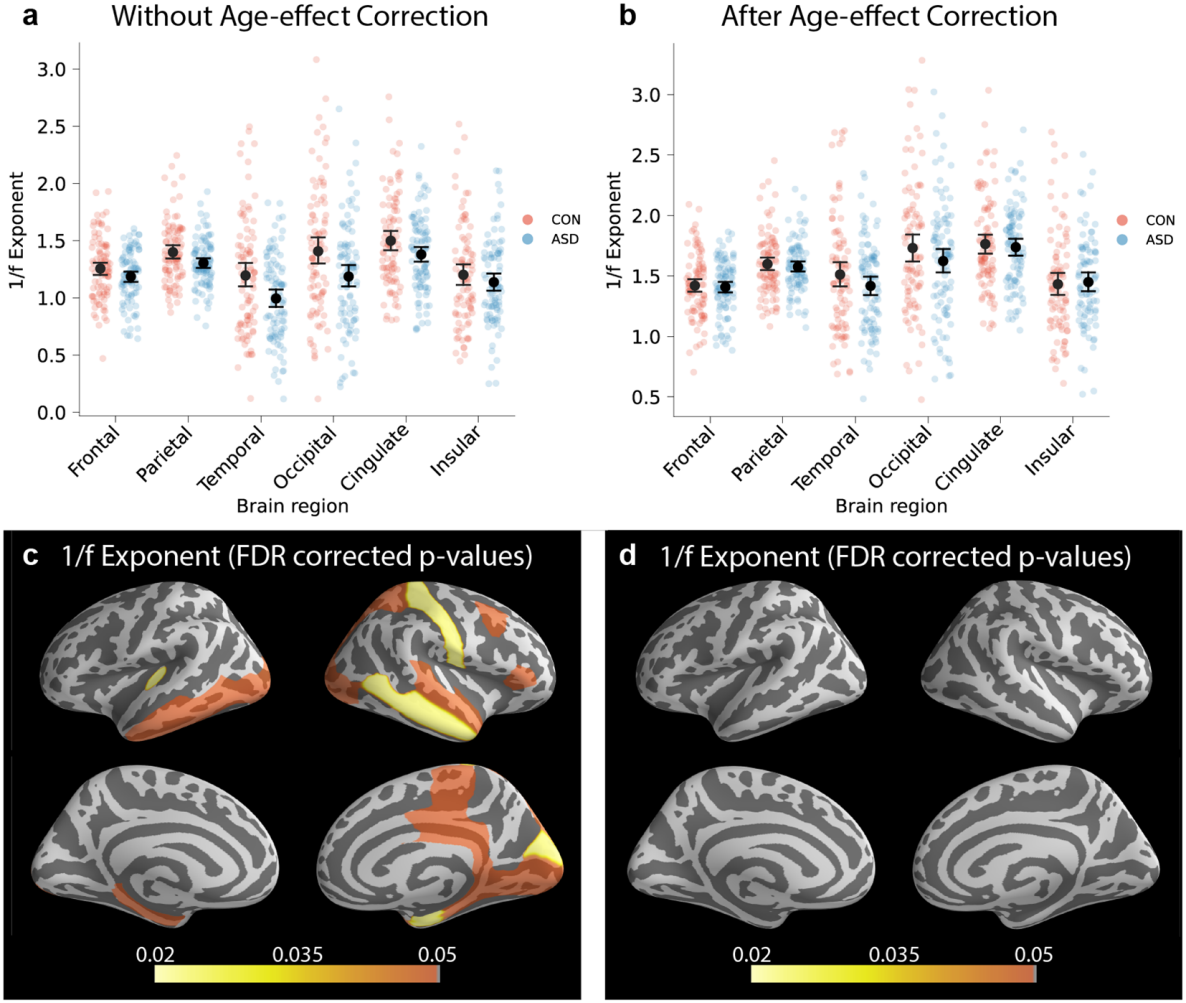
The effect of age on 1/f exponents. (**a**,**c**) Prior to age-effect correction of 1/f exponents, we observed a significant difference in group-means. (**b**,**d**) However, this effect was confounded by age, as evident by the lack of significant effects after we corrected for the age-effect. Mean with 95% confidence intervals are shown.

The importance of correcting for age-related effects can be observed when evaluating the mean differences in 1/f exponents between the ASD and non-autistic comparison group before and after age correction. Without age-effect correction, the 1/f exponents were significantly lower in the ASD group (Fig. 3a,c). However, this effect was confounded by the negative correlation between age and 1/f exponents (Fig. 2c), which becomes apparent after age-effect correction, where no significant effects were observed in 1/f exponents between the ASD and non-autistic comparison group (Fig. 3b,d).

### The ASD and non-autistic comparison group did not differ in group-mean or group-variability

**Figure 4:**
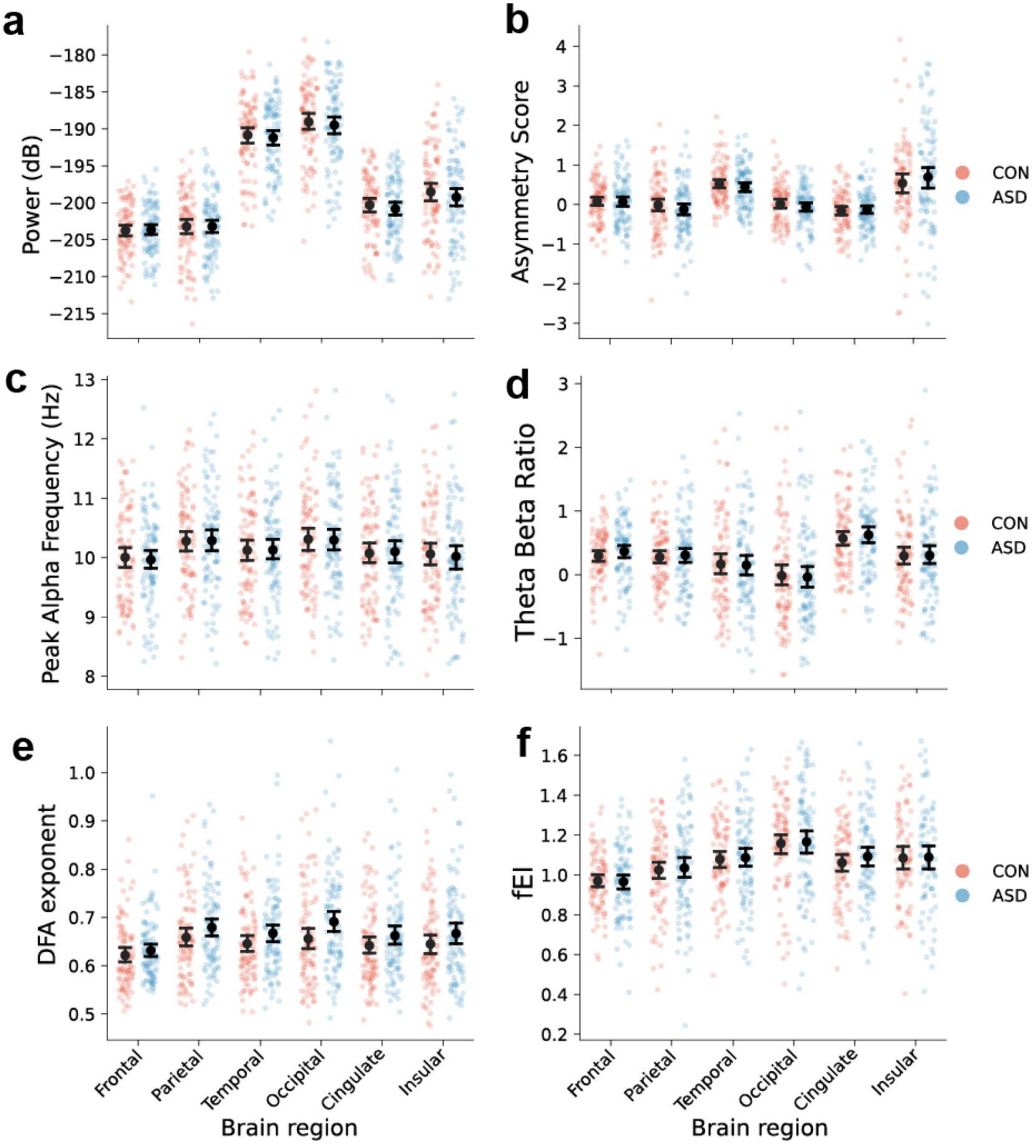
The ASD and non-autistic comparison groups have similar quantitative EEG features. Representative examples of computed EEG feature values averaged across the brain regions for: (**a**) Absolute Alpha Power, (**b**) Alpha Asymmetry, (**c**) Peak Alpha Frequency, (**d**) Theta/Beta Ratio, (**e**) Alpha DFA exponents, and (**f**) Alpha fEI. Mean with 95% confidence intervals are shown.

**Figure 5:**
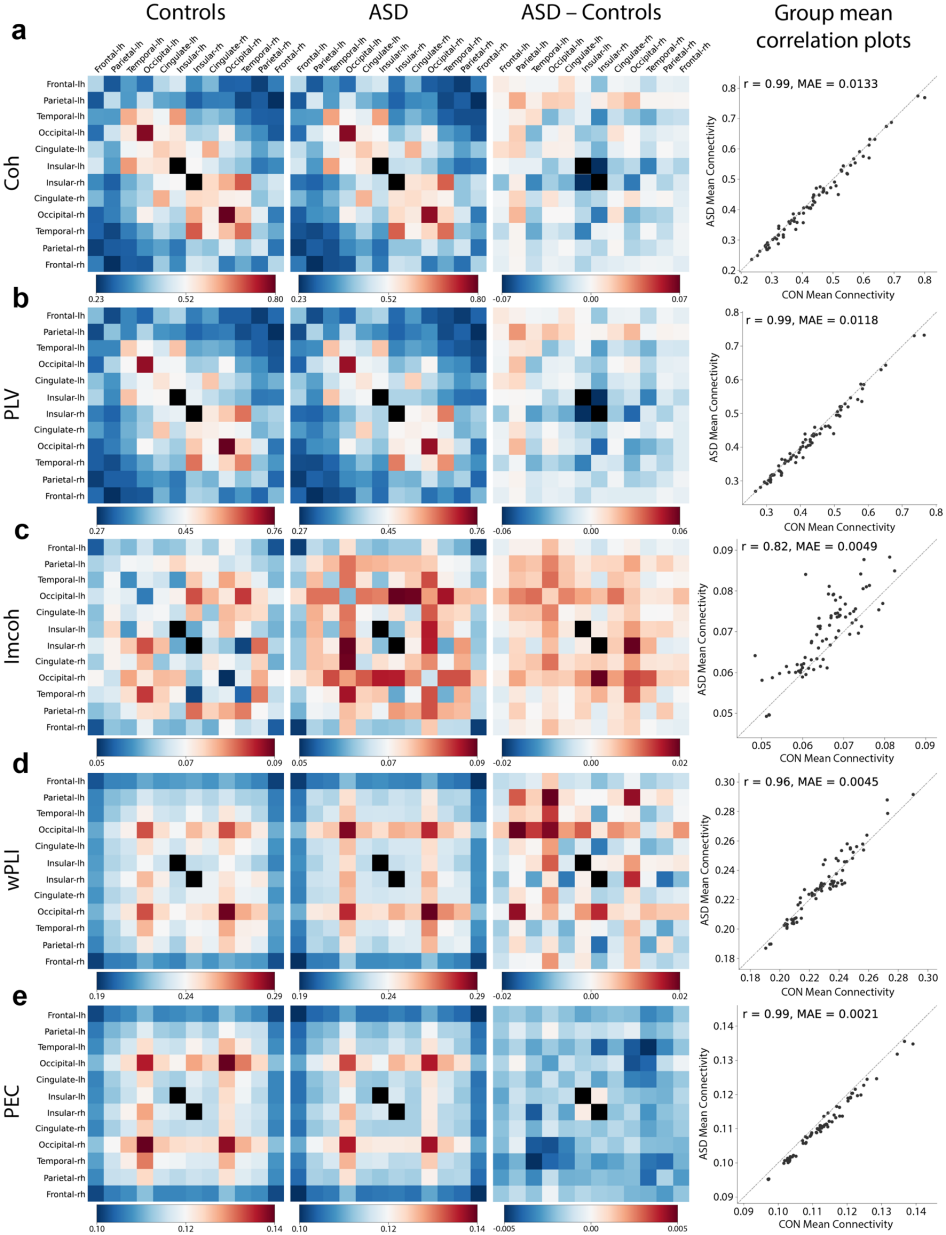
The ASD and non-autistic comparison groups have similar connectivity patterns. Examples of the mean (**a**) Coherence, (**b**) Phase Locking Value, (**c**) Imaginary Coherence, (**d**) Weighted Phase Lag Index, and (**e**) Power Envelope Correlations for the ASD and non-autistic comparison group, their difference, and correlation in the alpha band. The closer the points are to the diagonal line in the correlation plots, the better the correlation between the group-mean connectivities. Self-connectivity was excluded, hence the insular regions, which only contain one patch in each hemisphere, are colored black to reflect the missing value (see “Methods”). Pearson’s correlation was used to compute *r*. MAE, mean absolute error.

No EEG features differed significantly between the ASD and non-autistic comparison group after age-effect correction (Figs. 4 and 5). We performed permutation tests with FDR correction for each feature type separately, but did not observe any significant difference between the two groups ($p>$ 0.05 for all features).

Considering the large variation in symptomatology in ASD, it is plausible that the ASD and non-autistic comparison groups would show differences in variability in spite of the lack of mean differences. However, using Levene’s test with FDR correction for each feature type separately, we did not observe any significant difference in variability between the two groups ($p>$ 0.05).

### Machine learning models based on EEG features predicted ASD around chance-level

The statistical tests indicated that each individual EEG feature was not significantly different between the two groups. To investigate whether combinations of features could potentially serve as biomarkers for prediction of ASD, we employed multivariate machine learning models. To estimate how well the models would potentially predict on new unseen subjects, we trained and tested using two-layer cross-validation. The data was divided into a training set, which the model was trained on, a validation set, which was used to tune the hyperparameters, and a test set, which was the unseen data that was used to estimate the generalization performance of the models. Although we obtained decent training and validation accuracies, the performance dropped to around chance-level for most of the models when tested on completely unseen data. The best full classifier, which had access to all the features, was logistic regression with L1 regularization with a balanced test accuracy of 50.0%. None of the four full classifiers performed better than chance.

**Figure 6:**
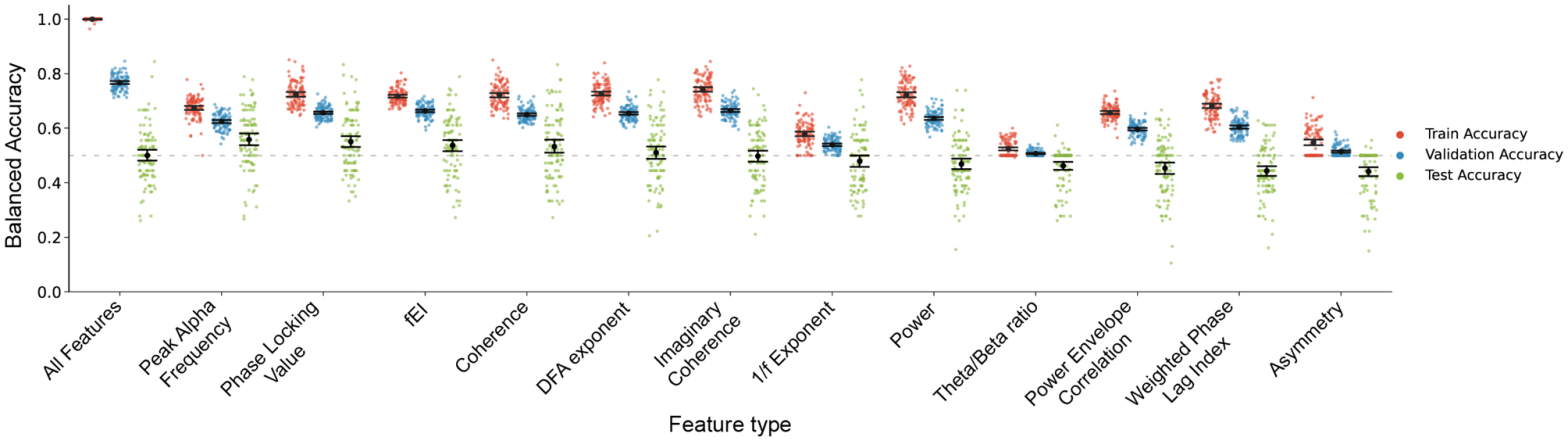
Performance of each feature type with logistic regression with L1 regularization. The logistic regression with all features are shown on the left, followed by the performances of each individual feature type, sorted according to average balanced test accuracy (green points). The dashed line indicates chance-level performance. Most of the feature types displayed significant training (red points) and validation (blue points) accuracies, which did not survive when tested on completely unseen data (cf. Fig. 7). Mean with 95% confidence intervals are shown.

We also tested each feature type separately, i.e., the same four classifiers were applied, but the feature selection was limited to each individual feature type. Here, the best classification performance was obtained by logistic regression with L1 regularization using peak alpha frequency with a balanced test accuracy of 55.8% (Fig. 6), followed by PLV with a balanced test accuracy of 55.1% and fEI with a balanced test accuracy of 53.7%. All three feature types performed significantly better than chance (one-sided Wilcoxon signed-rank test $p<$ 0.05), albeit the effects were small. Additionally, the performance of both peak alpha frequency and PLV was significantly better than chance-level in three out of the four classifiers, while fEI performed better than chance-level in two out of the four classifiers (See Supplementary Table S1 for the performance of all combinations of features and classifiers).

### Mean-level predictions of AQ and SPQ

Besides classifying ASD, we also investigated if combinations of EEG features would be able to predict the autism-spectrum quotient short questionnaire (AQ) score and the visual and auditory scores of the sensory perception quotient short questionnaire (SPQvis and SPQaud) using linear regression. The best full model was linear regression with L1 regularization, which obtained a normalized mean absolute error (nMAE) of 1.051 for AQ. None of the models with access to all features obtained a nMAE below 1, hence they did not perform better than predicting the mean on any of the questionnaire scales. We also tested each feature type separately and found the best performance for linear regression with L1 norm using fEI, which obtained a test nMAE of 0.960 for prediction of AQ. This result was significantly better than mean prediction (one-sided Wilcoxon signed-rank test p < 0.05), suggesting that fEI variability may be related to autism severity. However, no significant correlations between fEI and AQ were found after FDR correction, and the prediction was merely 4% better than the mean prediction MAE. The only other feature type with better than mean-level predictions was the theta/beta ratio, which obtained a test nMAE of 0.993 using linear regression with L1 regularization, and a test nMAE of 0.979 using linear regression with L2 regularization for AQ prediction. All other features did not perform better than predicting the mean on any of the questionnaire scales (see Supplementary Table S2 for the performance of all combinations of features, regression models, and target variables).

## Discussion

In spite of many attempts to identify EEG correlates of ASD, there is a lack of consistency of measures used and findings reported (for reviews, see^2,3,22^). With the aim of finding something more robust, we recruited, to our knowledge, one of the largest single-center adult ASD cohorts, and tested a comprehensive set of EEG features and machine learning models using two-layer cross-validation. This approach reinforced the picture that intellectually able adults with ASD show remarkably typical resting-state EEG. No significant group-mean or group-variance differences were observed between the ASD and non-autistic comparison group across the 3443 features investigated after FDR correction within biomarker types, which is in agreement with the visual inspection of biomarkers showing only minute differences in means (Fig. 4). Correspondingly, our machine learning models only performed slightly better than chance-level.

One caveat with an exploratory analysis involving many different features is the need for multiple testing correction, which might have resulted in a relatively lower statistical power compared to studies conducted in a hypothesis-driven paradigm. However, to not be too conservative and be more relatable to the other resting-state EEG studies, we only corrected for the number of features within each feature type separately, and even with this consideration, we still did not achieve any significant group-mean or group-variance differences. Visual inspection of distributions of EEG feature values (e.g. Fig. 4) also did not invoke the impression that the lack of significance is due to harsh multiple comparison correction.

Furthermore, the primary aim of our study was not to identify group-mean differences within single features, but to combine the many different features, and utilize machine learning to infer which combinations of features might have potential value as biomarkers for ASD. Even if each individual feature has small effect sizes, they might still provide relevant predictive information if they are combined in a multivariate model^40^. However, our machine learning models were unable to find an EEG pattern characteristic for the ASD group.

This is inconsistent with some of the studies using machine learning on resting-state EEG in ASD which reported classification accuracies above 87%^15,16,24–31^. However, the evaluation of the model performances were problematic in some of the studies. Two studies^10,33^ did not perform any cross-validation, which means they trained and tested on the same data and this has the caveat of the models overfitting to the data, which would result in the models being unable to generalize to new data^36–39^. Thus the reported performance of their models would correspond to the high training accuracies we observed (e.g. the red points in Fig. 6). The other studies employed cross-validation, but some performed feature selection on the whole data before splitting the data^30,31^, which means information leakage might have occurred, and the reported classification performances might be over-estimated. Another potential information leakage problem was observed in a study that split the data based on epochs, hence the same subjects might occur in both the training and test sets^25^. Some studies also only employed a single layer of cross-validation, despite having hyperparameters that should be tuned^26,27,29,31,35^. If a single layer of cross-validation is used for training and tuning, then there is no test set to estimate how well the model would generalize to new unseen subjects (reviewed in^37^). It would correspond to the validation accuracies we observed, where we obtained more than 80% accuracy in multiple folds using the model with all features (e.g. the blue points in Fig. 6).

Nonetheless, it is also important to stress that a single layer of cross-validation is enough if no hyperparameters are tuned, e.g., if the models do not have hyperparameters^16,24,32^, or if default values for hyperparameters are selected a priori (reviewed in^37^). Since our models had hyperparameters, we employed two-layer cross-validation to obtain a robust estimation of the predictive performance of our machine learning models. The hyperparameters and number of features were tuned in the inner layer, and we tested the generalization performance in the outer layer. Surprisingly, although we obtained decent validation performances with some models achieving above 80% validation accuracies, there was a large drop in performance when we evaluated on entirely unseen data, to the point where our best models merely performed slightly better than chance-level, further highlighting the importance of rigorous model evaluation. This finding is consistent with a recently published EEG study on ASD that obtained up to 57% balanced accuracy for ASD, when testing on unseen data using a nested cross-validation scheme^34^. Interestingly, we observed a high variance in test accuracies, reflecting how big an impact the exact split of the data could have on our machine learning models.

One of the most common arguments for inconsistent results across different studies is that methodological differences confound the results. However, the high variation we observed in test accuracies suggest that even if the facilities, equipment, preprocessing steps, algorithms, and researchers are the same, the results can still vary substantially based on the specific participants in the sample. Specifically, we obtained performance values ranging from not better than chance-level up to around 80% balanced test accuracy on classifying ASD. This clearly highlights the highly heterogeneous nature of ASD, and combined with differing recruitment methods, facilities, analysis methods and relatively small sample sizes in clinical ASD EEG studies, it is not surprising that inconsistent findings occur, and that robust neural biomarkers have not yet been found for ASD. The high variance in test accuracies also stress the importance of rigorous model evaluation schemes, e.g. two-layer cross-validation for models with hyperparameters^37^, for identification of robust biomarkers.

The problem with small sample sizes not being representative of the clinical population is also clearly illustrated by the paradox that machine learning models seem to perform worse on more neuroimaging data^23,41^. This negative correlation with sample sizes does not mean that the models trained on more data are worse, but instead that with few samples the models are more likely to overfit to bias within the sample, that is not reflective for the whole clinical population. Thus, subtle within group differences might be misinterpreted as between group differences. Additionally, this issue might be further inflated by publication bias^42^.

Apart from methodological differences, there are theoretical considerations that should be taken into account as well. The definition of ASD has become a topic of interest recently for three reasons: Firstly, ASD prevalence has more than tripled in recent years, from 0.67% in the year 2000 to 2.3% in 2018^43^. Secondly, the criteria for ASD according to the DSM has changed considerably in the past decades, most prominently between its fourth and fifth edition where multiple previously distinct conditions have been merged into the current condition “Autism Spectrum Disorder”. Thirdly, it has been shown that psychological and neurological effect sizes in ASD research have been continually decreasing in the past decades while no such effect was shown for schizophrenia research^44^. While the increase in ASD prevalence could be attributed to an increased public awareness or other external factors such as pollution^45^, it can also partly be attributed to ASD being used for a broader spectrum of symptoms and disorders. The increased heterogeneity could partly explain the decreased effect sizes in ASD research, since higher heterogeneity would result in increased within group variance^46^. Our results also support the notion of high heterogeneity in ASD.

We acknowledge that the present study has limitations. The machine learning models were trained on commonly used EEG features and are thus dependent on these specific feature types. We developed a comprehensive framework to encompass a broad number of feature types to investigate whether each feature or combinations of features could characterize ASD, but other combinations of EEG features remain unexplored, e.g., graph theory based network metrics, entropy, and microstates have also been associated with ASD^47–49^.

Another limitation to the present study is the experimental paradigm. We investigated the EEG activity during 5 min eyes-closed rest, which might be different from eyes-open rest or task-based paradigms^50^. One previous study found group differences in alpha power and coherence during eyes-open EEG in adults with ASD, but not in the eyes-closed condition^13^.

The study sample should also be highlighted. The ASD group is comprised of adults with average or above average intelligence, often employed, late-diagnosed and well educated, while many previous ASD EEG studies focused on children. Therefore, discrepancies between our results and studies investigating children with ASD might occur; e.g., in a previous study, we observed that children with ASD had greater group-mean DFA and higher variability in DFA and fEI^20^, but this result was not observed in the sample investigated in this study. One might argue that because many individuals in the ASD group were not diagnosed at an early age, they might have less pronounced autistic traits or been better at compensatory strategies, however, we did not see an association between AQ score and age of diagnosis (Supplementary Fig. S1). AQ is a self-report measure of autistic trait, but the only quantitative measure of autism severity available uniformly from all participants.

It should also be highlighted that both our ASD and non-autistic comparison group included 57% and 54% females, respectively. This is higher than the general autistic population, which has a strong male bias. The participants in this study were recruited based on voluntary sign-up and, historically, females volunteer more readily for such activities. Although our female bias might make the results less relatable to other studies with a strong male bias, there were also benefits of having a relatively gender balanced dataset. The machine learning models that we trained would be less likely to be better at classifying males relatively to females due to discrepancies in number of training samples.

Overall, our results indicate that intellectually able adults with ASD have eyes-closed resting-state EEG activity within the typical range. This does not necessarily mean that resting-state EEG traces contain no meaningful information about ASD, however, the electrophysiological effects were likely too subtle to be picked up by our models relative to the high heterogeneity in ASD, even with a sample size of close to 200 participants. Future studies should try to increase effect sizes or mitigate the effect of heterogeneity by increasing sample sizes to better represent the whole ASD population and/or look for prototypes^46,51^ or distinct subtypes within ASD. Identification of subtypes might improve diagnosis and enable better tailored treatment on an individual/subgroup level^23,41,52^. Differences might also be more prominent during specific tasks targeted at the autistic traits, e.g. crucial differences could be picked up by employing interactive paradigms, and studying interpersonal mechanisms in ASD^53–58^. Encompassing longitudinal data or data from multiple modalities might also increase effect sizes^40^, e.g., combining genetics (heritability was estimated to be around 83% for ASD^59^), neuroimaging, psychological, and social information. The analysis framework we developed can be readily expanded to integrate with all of these modalities and support the development of a biopsychosocial model for ASD^60,61^.

## Methods

### Participants

The participants were part of a larger project investigating mechanisms underlying ASD in adults and recruited through the Netherlands Autism Register (NAR^62^). Upon registration, autistic adult participants of the NAR confirmed that they have a clinical diagnosis of autism spectrum disorder (ASD) as established by an authorized professional (e.g., psychiatrist/psychologist) based on Diagnostic and Statistical Manual of Mental Disorders (DSM-IV^63^ or DSM-5^1^). Specifically, inclusion criteria for the ASD cohort were a clinical diagnosis of ASD (according to DSM-5), Asperger’s syndrome, pervasive developmental disorder-not otherwise specified, autism (according to DSM-IV), and age between 18 and 55 years. Exclusion criteria for the non-autistic comparison group was a diagnosis of ASD or a diagnosis of ASD in a direct family member. The clinical professionals worked independently from the authors, and who were unaware of the goals and outcomes of this study. The diagnostic process included anamneses, proxy reports, and psychiatric and neuropsychological examinations. Individuals have to confirm their autism diagnosis and disclose additional information on when, where, and by whom (psychiatrist/psychologist) the diagnosis was determined, as well as the measure used (ADOS or ADI-R)^64^. Moreover, official proof of autism diagnoses could be obtained for a subsample of individuals in the NAR. Patterns of functioning in this group were similar to the rest of the sample^64^. Additionally, previous findings on the validity of parent-reported autism diagnosis to a web-based register supports the validity of the register diagnosis^65^. Of the autistic participants, 53% had paid employment, 41% were married or in a relationship (7% were divorced), 7% lived with their parents or in residential care. A more detailed description of the adult participants of the NAR can be found in Scheeren et al. 2022^66^. Table 2 presents the characteristics of the included participants. The protocol of this study was approved by the ethics committee of the VU University Medical Center (approval number 2013/45). The study was conducted in accordance with the guidelines and regulations approved by the respective ethical committee and in compliance with the provisions of the declaration of Helsinki. All participants provided informed consent and were financially reimbursed. Data and scripts from this study are available upon request.

**Table 2:** Characteristics of study participants.

	CON	ASD
Sample size	91	95
Mean age (SD), years	32.34 (12.25)	43.65 (8.99)
Sex (female), %	57.1	53.7
Mean AQ (SD)	50.97 (10.05)	85.73 (9.88)
Mean auditory SPQ (SD)	9.13 (2.51)	6.66 (2.51)
Mean visual SPQ (SD)	10.54 (2.98)	7.07 (3.15)

*AQ* Autism-spectrum quotient, *SPQ* Sensory Perception quotient, *SD* standard deviation

### Clinical measures

#### Autism-spectrum quotient short questionnaire

The autism-spectrum quotient short questionnaire (AQ^67^) is an abridged version of the autism-spectrum quotient^68^. It consists of 28 self-report items, which can be clustered into two main factors: Social Behavior, and Numbers and Patterns. The items under Social Behavior can further be divided into the subfactors: Social Skills, Routine, Attention Switching, and Imagination. All items are scored on a four-point Likert scale and range from “Definitely agree” to “Definitely disagree”. The items are summed to obtain total AQ scores. Higher AQ score suggest more autistic traits.

#### Sensory perception quotient short questionnaire

The sensory perception quotient short questionnaire (SPQ^69,70^) is a 35-item self-report assessing sensory perception of touch, smell, vision, hearing, and taste. We only used the items pertaining to the factors vision (6 items) and hearing (5 items). All items are scored on a four-point Likert scale and range from “Strongly agree” to “Strongly disagree”. The items are summed to obtain factor scores (SPQvis and SPQaud). Lower scores on the SPQ suggest a lower sensory threshold, thus higher sensory sensitivity.

### EEG acquisition

Resting-state EEG was recorded during 5 min of eyes-closed rest with a 64-channel BioSemi system sampled at 2048 Hz. The participants received the instructions: “Please keep your eyes closed, relax, and try not to fall asleep”. Impedance across all electrodes was kept below 5 k$\Omega$. Additionally, four electrodes were placed at the left and right outer canthi to capture horizontal eye movements, and an electrode underneath each eye for vertical eye movements and blinking.

### EEG pre-processing

The EEG data were processed using MNE-Python 0.24.3^71^. First, the data were band-pass filtered at 1–100 Hz, notch filtered at 50 Hz, downsampled to 500 Hz, and divided into 4 s epochs without overlap. Bad epochs and channels with gross non-ocular artefacts were rejected by visual inspection. The data were re-referenced to the common average and ocular and ECG artefacts were removed using Piccard independent component analysis^72^ with the number of components set to 32. Autoreject 0.2.2^73^ was employed to catch any remaining artefacts, which guided a final visual inspection. Subjects with less than 2 min of clean signal were excluded from further analysis (*n* = 3), resulting in a total sample size of 95 ASD and 91 non-autistic participants. The EEG features were estimated in the five canonical frequency bands: delta (1.25–4 Hz), theta (4–8 Hz), alpha (8–13 Hz), beta (13–30 Hz), and gamma (30–48 Hz), unless otherwise stated.

### EEG source localization

We used L2 minimum norm estimation, as implemented by MNE-Python, to obtain cortical current estimates from our sensor level data. The FreeSurfer average brain template from FreeSurfer 6^74^ was used to construct the boundary element head model and forward operator for the source modelling. The regularization parameter was set to $\lambda ^2=1/9$. A diagonal matrix with 0.2 V values was used for the covariance matrix, which was the default values for EEG provided by MNE-Python. Unconstrained orientations were allowed, and principal component analysis was employed on the whole source time series at each vertex to reduce the three-dimensional signals to one-dimensional time series of the dominant principal component. The time series of the 20484 source vertices were further collapsed into 68 cortical patches based on the Desikan Killiany atlas, by first aligning the dipole orientations by shifting vertices with opposite polarity to the majority of vertices by $\pi$, followed by averaging the amplitudes of all vertices within a patch. The phase shifting prevents the vertices with opposite polarities from canceling each other out during the averaging operation. Some of the estimated EEG features were computed across cortical brain regions, i.e., patches within frontal, parietal, temporal, occipital, cingulate, and insular regions were averaged. The individual patches were mapped to the brain regions according to the appendix in Klein et al., 2012^75^.

### EEG feature estimation

A brief description of each EEG feature type is provided (see Fig. 1 for overview of all features), as all the estimated features are already well-established. More detailed information and equations can be found in the supplementary information.

#### Spectral analysis

Multitaper spectral estimation^76^ was employed to estimate absolute and relative power in the five canonical frequency bands. EEG power asymmetry was calculated by subtracting each left hemispheric patch from the corresponding patch in the right hemisphere^77^, followed by averaging across the brain regions. Theta/beta ratio was computed by dividing theta power by beta power and averaged in brain regions. Peak alpha frequency and 1/f exponent was estimated using the FOOOF algorithm^78^. In total we estimated 680 power features, 6 theta/beta ratios, 30 asymmetry features, 69 peak alpha frequency features, and 68 1/f exponents.

#### Criticality

Long-range temporal correlations^79^ were estimated following the detrended fluctuation analysis (DFA) procedure described in Hardstone et al., 2012^80^ to obtain DFA exponents. Functional excitation/inhibition ratio (fEI) was estimated according to Bruining et al., 2020^20^. In total we estimated 345 DFA and fEI features, one for each brain patch and a global averaged value in each of the five frequency bands.

#### Functional connectivity

All functional connectivity measurements were computed for all pairwise combinations of the 68 brain patches. This yields 2346 connections, which results in 56,950 features when taking into account all connections are calculated for every frequency bands and five different connectivity measurements. This vast number is many times higher than the number of subjects we have, which can lead to overfitting for the machine learning models (also known as curse of dimensionality^81^). To reduce the number of dimensions, we averaged the connectivity measurements across patches within brain regions and for each hemisphere, e.g., the connectivity between the frontal-lh and parietal-rh brain regions were computed as the average connectivity between all the brain patches located in the frontal-lh with all the brain patches located in the parietal-rh. When computing intra-regional connectivity, e.g., frontal-lh with frontal-lh, we again computed the average connectivity between all the brain patches located with the frontal-lh, but excluded self-connectivity for patches, i.e., the same brain patch with itself was not considered. Specifically for the insular regions, which only contained one brain patch, no intra-regional connectivity was estimated. In total we reduced the number of connections to 76, which across the five frequency bands amount to 380 features for each of the five connectivity feature types, resulting in a total of 1900 features.

Coherence (Coh) was calculated as the magnitude of the cross spectrum between two signals divided with the square root of the product of each signal’s power spectrum for normalization^82^. The imaginary part of coherence (Imcoh) was also used as a standalone feature, due to its lower sensitivity towards volume conduction^83^. We also computed the phase locking value (PLV), which measures connectivity as a function of phase difference variability^84^, and weighted phase lag index (wPLI), which also measures phase synchronization but is less sensitive towards volume conduction^85^. Lastly, power envelope correlations (PEC) were estimated following Toll et al., 2020^86^.

### Prediction

**Figure 7:**
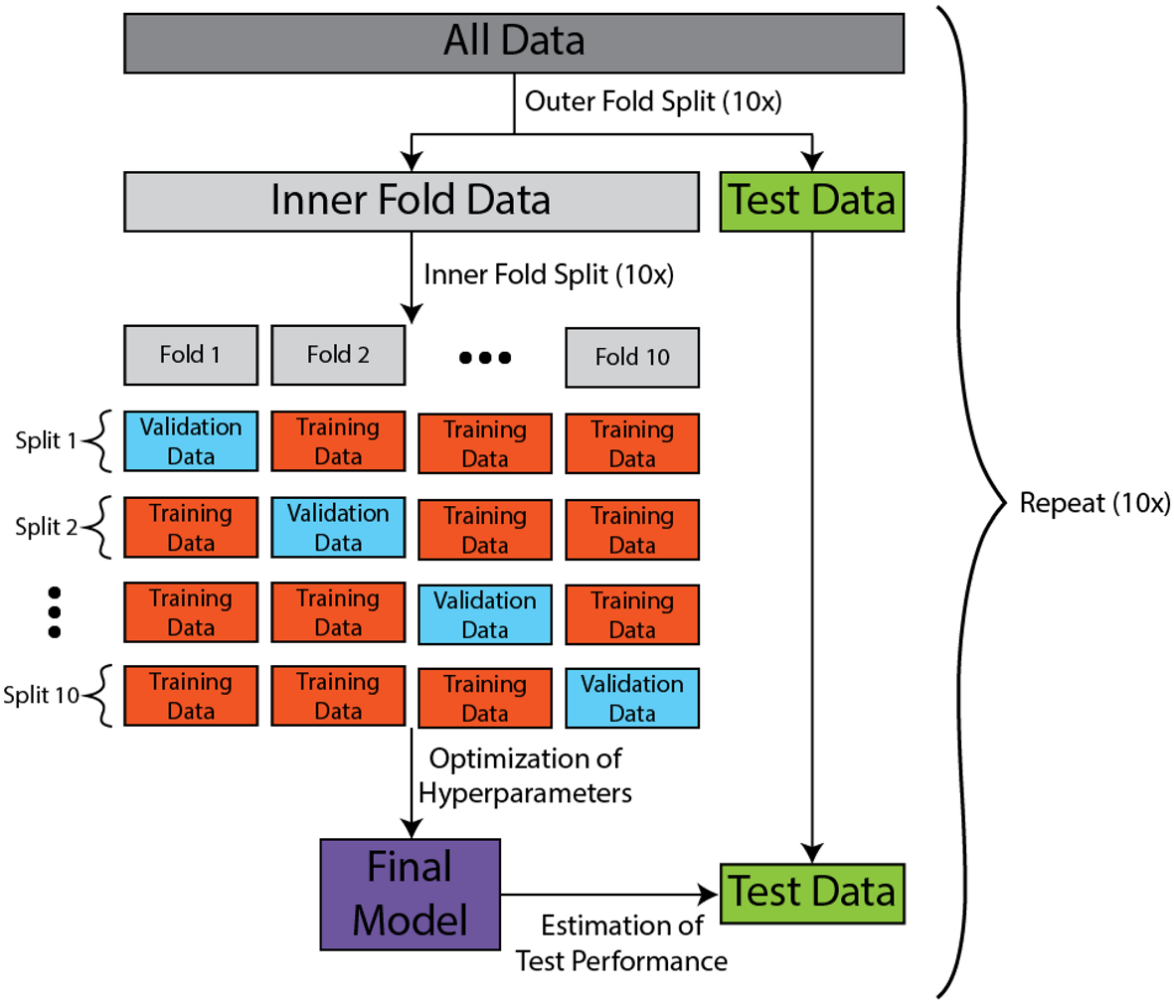
Schematic of the repeated two-layer cross-validation scheme. In order to obtain robust and reliable estimations of how well the machine learning models would perform on unseen data, we employed 10 repetitions of 10-by-10 two-layer cross-validation. The outer fold split ensured we tested on unseen data, while the inner fold split alleviates overfitting when we optimized model hyperparameters, i.e., regularization strength for logistic regression and SVM, number of trees and depth for random forest, and the number of features used by the models after feature selection.

All the EEG features were combined and turned into a data matrix of number of subjects by 3443 features. Given that there were more features than samples, and in order to decrease the dimensionality and reduce overfitting^81^, we applied multiple dimensionality-reduction methods. First, minimal-redundancy-maximum-relevance (mRMR^87^) was applied to each EEG feature type to attenuate the effect of imbalance in the number of features between each of the EEG feature types. To not have our results be dependent on one particular machine learning model, we trained variations of three commonly used machine learning models to predict ASD: (1) support vector machine (SVM) with recursive feature elimination, (2) logistic regression with Ridge regularization (L2) and sequential forward selection, (3) logistic regression with Lasso regularization (L1) and (4) random forest. For prediction of questionnaire scores, we employed linear regression with either L1 or L2 norm. Model training, hyperparameter tuning and evaluation of the models were conducted in a stratified 10-by-10 fold two-layer cross-validation scheme repeated 10 times to take into account the effect of random splits, resulting in 100 final models (10 repetitions of 10-fold outer cross-validation; Fig. 7). The stratification ensured the proportion of people with ASD were balanced across folds. Balanced accuracy, the mean of sensitivity and specificity, was estimated for the classifier performances. A balanced accuracy around 50% indicates chance-level prediction for a two-class classification. Normalized mean absolute error (nMAE), the ratio of MAE of the model to the MAE obtained when predicting the mean value of the training set, was computed for evaluation of the regression models. A nMAE = 1 indicates prediction around the level of just predicting the mean, while a lower nMAE indicates superior performance compared to predicting the mean value.

All model hyperparameters were tuned in the inner cross-validation fold on the validation set with grid search. Specifically, we tested the performance of using 10, 20, 30 or 40 features in mRMR, 16 values on a logarithmic scale from 0.01 to 1000 for the regularization parameter *C* for logistic regression, 13 values on a logarithmic scale from 0.001 to 1 for SVM, and 10, 50, 100, 500 or 1000 trees with depths 1 or 2 in random forest. The range of the hyperparameters were determined during preliminary analysis of when the models started to overfit and the step sizes were designed with the computational time in mind. We used mlxtend 0.19.0^88^ for sequential forward selection, scikit-learn 1.0.1^89^ for the implementation of cross-validation and the machine learning models, and a python implementation of mRMR^90^.

### Age-effect correction

It is well-established that some EEG features have an age-dependent effect. Both peak alpha frequency^91^, theta/beta ratio^92^ and 1/f exponents have been found to be lower in old compared to young adults^78,93–95^. DFA exponents have also been found to increase from childhood into early adulthood before it stabilizes^96^. Although the age range of both groups fell between 19 and 55 years, the ASD group was, on average, 11 years older than the non-autistic comparison group (Permutation test, $p =$ 0.0001). We investigated the linear correlations between each EEG feature type and age. For all the features that showed more significant Pearson’s correlations than chance-level after false-discovery rate (FDR) correction, we corrected for the age-effect by removing the linear trend. The FDR correction was applied to each feature type separately and the detrending was performed for the whole cohort.

### Statistical analysis

Results are shown as mean with 95% confidence intervals. A non-parametric permutation test with FDR correction was used to test for group mean differences. Levene’s test with FDR correction was used to assess equality of variances. The FDR correction was applied to each feature type separately. Pearson’s correlation coefficient and Student’s *t*-distribution was used to test for significant linear correlations. Non-parametric Wilcoxon signed-rank test was used to test differences between classifiers. One-sided tests were used for baseline comparisons (chance-level for the classifiers or mean prediction for the regressors). A p-value < 0.05 was considered significant for rejection of the null hypothesis.

## Supplementary Information

Supplementary Information.

## Data Availability

Data are available from SB and the code is available from QL on request.
