# Supplementary material for: Intellectually able adults with autism spectrum disorder show typical resting-state EEG activity
Source: Sci Rep. 2022 Nov 8;12:19016. doi: 10.1038/s41598-022-22597-z (PMC9643446; doi:10.1038/s41598-022-22597-z)
Supplement: Supplementary file 1 — Supplementary Information. [file 41598_2022_22597_MOESM1_ESM.pdf]

Supplementary materials

Supplementary methods

Detailed information about how each individual EEG feature was computed can be found here.

Power

EEG power was computed using multitaper spectral estimation with 7 discrete prolate spheroidal sequences (DPSS) windows for the five canonical frequency bands. Two transformations were applied to the power features; absolute power was converted into decibels (dB) by taking the log base 10 and multiplying with 10, and relative power was computed as proportion of power in each frequency band normalized to the total power in all frequency bands (1.25–48 Hz).

Asymmetry

EEG power asymmetry was computed as the log transformed raw power (i.e., prior to absolute/relative transformation) in each left hemispheric patch subtracted from the corresponding patch in the right hemisphere (1):

$$\text{Asymmetry} = \ln(Power_{rh}) - \ln(Power_{lh}) \tag{1}$$

The asymmetry values were averaged across the brain lobes to yield frontal, parietal, temporal, occipital, cingulate, and insular asymmetry for each of the five frequency bands.

Theta/beta ratio

Theta/beta ratio was estimated by taking the averaged raw theta and beta power in the six brain lobes followed by computing their ratio and applying the natural logarithm.

Peak alpha frequency and 1/f exponent

Peak alpha frequency and 1/f exponent was estimated using the FOOOF algorithm (2). The FOOOF algorithm assume the power spectral density is composed of the aperiodic (1/f) component and periodic components (e.g., alpha oscillations). A power law is used to fit the aperiodic component, while multiple Gaussian fits are utilized to fit the periodic components. We used the R-squared of the fit of the full model to evaluate how well the algorithm worked. Based on visual inspection of the fits and the R-squared values, we decided that for our data the peak alpha frequency estimations of models with an R-squared > 0.90 was reliable. For the 1/f exponents, we set the thresholds to be R-squared > 0.95. If the R-squared was lower than the threshold, the peak alpha frequency or 1/f exponent values were set to NaN. Due to the presence of delta/theta peaks, which were harder to fit due to their location on the lower end of the power spectra, the R-squared values might be below the threshold, despite the fit being good. To circumvent this specific problem, we iteratively tried fitting the FOOOF algorithm using 2, 3, 4, 5 or 6 Hz as the lower frequency and if the R-squared was above the thresholds then we used the estimated PAF and 1/f exponents. The end range of the fit was set to the end of the gamma-frequency range (48 Hz). Peak alpha frequency and 1/f exponents were calculated for each brain patch and a global peak alpha frequency was also computed as the average over all patches.

Long-range temporal correlations

Long-range temporal correlations were estimated following the detrended fluctuation analysis (DFA) procedure described by Hardstone et al, 2012 (3). Window sizes were equally spaced on a logarithmic scale and specified to be between 5 and 30 s. Specifically, the lowest window size was 5.54 s and the highest window size was 28.94 s. The DFA exponent indicates that the

signal exhibits long-range anti-correlations if DFA < 0.5, DFA exponent ~ 0.5 indicates it is indistinguishable from a random process (i.e. uncorrelated), while a DFA exponent > 0.5 indicates the signal exhibits long-range positive correlations. DFA exponents for each of the five canonical frequency bands were computed. Nolds 0.5.2 (4) was used to implement DFA.

Functional excitation/inhibition ratio

Functional excitation/inhibition ratio (fEI) was estimated following Bruining et al, 2020 (5), and as recommended we only estimated fEI if DFA > 0.6. Windows of 5 seconds with 50% overlap was used for the computation. Sub-critical networks have fEI < 1, critical networks have fEI ~ 1, while super-critical networks have fEI > 1. fEI for each of the five canonical frequency bands were computed.

Coherence

Coherence can be viewed as the frequency-domain analogue to Pearson's correlation in the time domain, and both measure the linear dependency of two signals. Coherence was calculated as the magnitude of the cross spectrum between two signals divided with the square root of the product of each signal's power spectrum for normalization (6).

$$\text{Coh} = \frac{|G_{xy}|}{\sqrt{G_{xx}G_{yy}}} \tag{2}$$

Here G_{xy} is the cross-spectral density between channels x and y and G_{xx} and G_{yy} is the power spectra of each signal respectively. A coherence value close to 1 indicates strong synchronization, while a value close to 0 reflects no synchronization between the two signals. Coherence values were calculated for each epoch and then averaged.

Imaginary Coherence

One problem with coherence is that it is sensitive to volume conduction, but by using only the imaginary part of coherence, the effect of zero-phase lag synchronization, which is the hallmark of volume conduction, can be removed (7).

$$\text{Imcoh} = \frac{Im(G_{xy})}{\sqrt{G_{xx}G_{yy}}} \tag{3}$$

The drawback of using imaginary coherence is that true non-volume conducted neuronal synchronization around zero-phase lag will also be attenuated, thus potentially leading to an underestimation of the true synchronization.

Phase Locking Value

The cross-spectral density contains information about both the amplitudes and the phase difference between the two signals of interest. When looking at the synchronization between two signals, using only the relative phase information and disregarding the amplitudes might be better at capturing the underlying neural activity as it becomes less sensitive towards noise. This is the idea behind the phase-locking value (8). The phase-locking value was originally defined as the inter-trial variability of the phase difference at time point t, which is appropriate when looking at event-related potentials (ERPs). However, for resting-state data, the variability of the phase difference cannot be calculated across trials, so here we calculate it over time.

$$\text{PLV} = \frac{1}{T}\left|\sum_{t=1}^{T} e^{i\Delta\phi_t}\right| \tag{4}$$

Where $\Delta\phi_t = \phi_{(x,t)} - \phi_{(y,t)}$ is the instantaneous phase difference in radians between the two signals of interest at timepoint t and T is the total number of timepoints within one epoch. If the phase differences are consistent over time, PLV is equal to 1, while if the phase differences are randomly distributed over time, PLV will be close to 0. The continuous wavelet transform

was used to decompose into frequencies. After estimation of PLV in each epoch the values were averaged to obtain a more robust estimation.

Weighted Phase Lag Index

A common source due to volume conduction might lead to consistent zero-phase differences and would thus inflate PLV. Thus, the phase lag index was developed to circumvent this problem, by calculating how consistent one signal lead/lag behind the other signal. This is based on the assumption that if one signal consistently leads the other signal, then there is also a consistent non-zero-phase difference, whereas volume conduction would lead to a symmetrical phase difference distribution around zero-phase (9).

$$\text{PLI} = \frac{1}{T}\left|\sum_{t=1}^{T} sign[\sin(\Delta\phi_t)]\right| \tag{5}$$

Similar to the idea behind imaginary coherence, the weighted phase lag index (wPLI) was developed to further attenuate the effect of volume conduction by weighing each phase difference with the magnitude of the lag, hence ensuring that phase differences around 0 will contribute minimally to the estimation of the connectivity (10; 11).

$$\begin{aligned} \text{wPLI} &= \frac{\frac{1}{T}\left|\sum_{t=1}^{T} |\sin(\Delta\phi_t)| \cdot sign[\sin(\Delta\phi_t)]\right|}{\frac{1}{T}\sum_{t=1}^{T} |\sin(\Delta\phi_t)|} \\ \Leftrightarrow \text{wPLI} &= \frac{\left|\sum_{t=1}^{T} |\sin(\Delta\phi_t)| \cdot sign[\sin(\Delta\phi_t)]\right|}{\sum_{t=1}^{T} |\sin(\Delta\phi_t)|} \end{aligned} \tag{6}$$

The denominator normalizes the wPLI to the interval $0 \leq \text{wPLI} \leq 1$, where higher values indicates more synchronization.

Power Envelope Correlations

Power envelope correlations (PEC) were estimated following Toll et al, 2020 (12). Briefly, the time series for each signal pair were bandpass filtered for the canonical frequency bands, Hilbert transformed to obtain the analytical signals and then orthogonalized to each other. The orthogonalization ensures that the signal components which share the same phase are removed, i.e. volume conduction induced zero-phase lag correlations are removed (13). Following orthogonalization, the power envelope of the orthogonalized analytical signals are estimated and log-transformed. Finally, Pearson's correlations were calculated and Fischer's r-to-z transform applied.

Supplementary figures

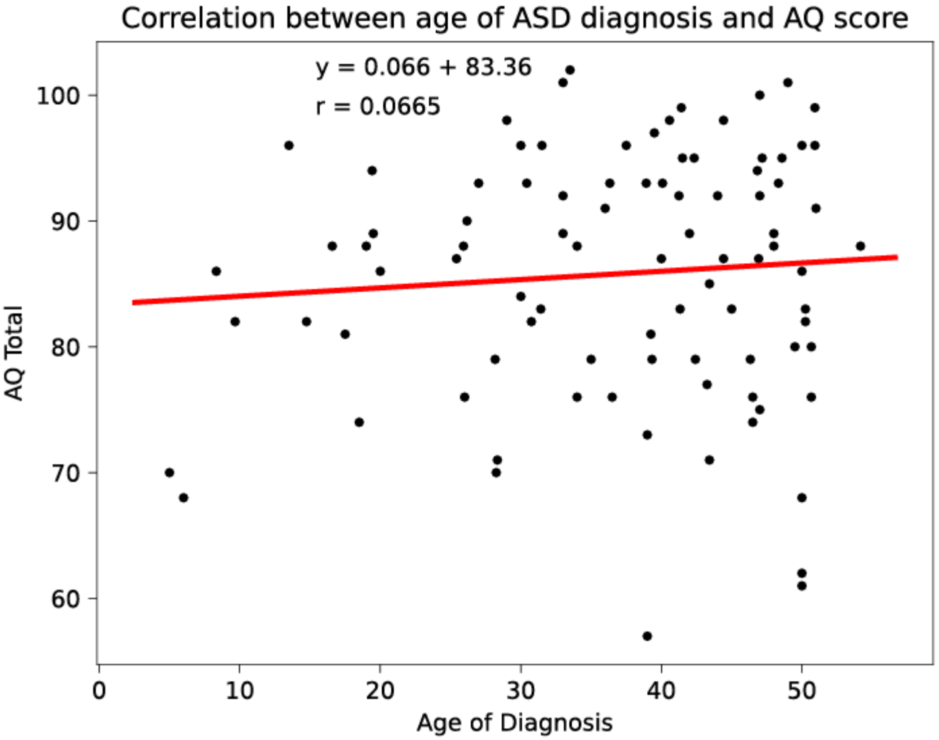

Figure S1. **Lack of correlation between age of diagnosis and AQ scores.** The AQ scores were not correlated with the age of ASD diagnosis.

Supplementary tables

Supplementary Table S1 contains the classifier performances for all combinations of machine learning models and feature types. Please see the corresponding Excel table.

Supplementary Table S2 contains the regression performances for all combinations of machine learning models and feature types. Please see the corresponding Excel table.

Supplementary Table S1: Classification performance for all combinations of models and features

Feature	Model	Training Accuracy	Validation Accuracy	Test Accuracy
All Features	Logistic Regression with L1 norm	1.000	0.767	0.500
	Logistic Regression with L2 norm	0.862	0.844	0.483
	Linear SVM	0.943	0.801	0.473
	Random Forest	0.945	0.694	0.494
Power	Logistic Regression with L1 norm	0.723	0.636	0.468
	Logistic Regression with L2 norm	0.731	0.718	0.451
	Linear SVM	0.729	0.670	0.476
	Random Forest	0.772	0.632	0.482
Theta/Beta Ratio	Logistic Regression with L1 norm	0.525	0.507	0.462
	Logistic Regression with L2 norm	0.551	0.546	0.459
	Linear SVM	0.528	0.518	0.454
	Random Forest	0.696	0.548	0.497
Asymmetry	Logistic Regression with L1 norm	0.548	0.514	0.442
	Logistic Regression with L2 norm	0.612	0.600	0.432
	Linear SVM	0.597	0.558	0.444
	Random Forest	0.723	0.562	0.498
Peak Alpha Frequency	Logistic Regression with L1 norm	0.675	0.625	**0.558**
	Logistic Regression with L2 norm	0.708	0.692	0.502
	Linear SVM	0.683	0.646	**0.541**
	Random Forest	0.761	0.613	**0.532**
1/f Exponent	Logistic Regression with L1 norm	0.579	0.539	0.479
	Logistic Regression with L2 norm	0.639	0.636	0.453
	Linear SVM	0.602	0.583	0.481
	Random Forest	0.669	0.565	0.482
DFA Exponent	Logistic Regression with L1 norm	0.727	0.654	0.510
	Logistic Regression with L2 norm	0.750	0.739	0.529
	Linear SVM	0.741	0.687	0.510
	Random Forest	0.766	0.648	**0.542**
fEI	Logistic Regression with L1 norm	0.717	0.663	**0.537**
	Logistic Regression with L2 norm	0.760	0.756	0.518
	Linear SVM	0.735	0.701	**0.538**
	Random Forest	0.794	0.659	0.525
Coherence	Logistic Regression with L1 norm	0.721	0.650	**0.533**
	Logistic Regression with L2 norm	0.738	0.724	0.509
	Linear SVM	0.730	0.680	**0.537**
	Random Forest	0.765	0.640	0.503
Imaginary Coherence	Logistic Regression with L1 norm	0.742	0.665	0.497
	Logistic Regression with L2 norm	0.765	0.748	0.501
	Linear SVM	0.754	0.699	0.500
	Random Forest	0.786	0.650	**0.526**
Phase Locking Value	Logistic Regression with L1 norm	0.724	0.656	**0.551**
	Logistic Regression with L2 norm	0.743	0.730	**0.543**
	Linear SVM	0.739	0.681	**0.539**
	Random Forest	0.765	0.627	0.487
Weighted Phase Lag Index	Logistic Regression with L1 norm	0.681	0.604	0.443
	Logistic Regression with L2 norm	0.707	0.695	0.435
	Linear SVM	0.697	0.635	0.441
	Random Forest	0.753	0.617	0.500
Power Envelope Correlation	Logistic Regression with L1 norm	0.657	0.596	0.453
	Logistic Regression with L2 norm	0.690	0.687	0.452
	Linear SVM	0.659	0.624	0.453
	Random Forest	0.735	0.620	0.483

Accuracy are mean balanced accuracy over 10 repetitions of 10-by-10 two-layer crossvalidation runs (100 outer fold runs). Numbers in **bold** indicate the performance was significantly better than chance-level (One-sided Wilcox test with FDR correction for the 12 feature types for a given model type)

Supplementary Table S2: Regression performance for all combinations of models and features

Target Variable	Feature	Model	Training Error	Test Error
Autism Quotient	All Features	Linear Regression with L1 norm	0.444	1.051
		Linear Regression with L2 norm	0.315	1.243
	Power	Linear Regression with L1 norm	0.833	1.097
		Linear Regression with L2 norm	0.768	1.182
	Theta/Beta Ratio	Linear Regression with L1 norm	0.966	**0.993**
		Linear Regression with L2 norm	0.957	**0.979**
	Asymmetry	Linear Regression with L1 norm	0.998	1.003
		Linear Regression with L2 norm	0.948	1.079
	Peak Alpha Frequency	Linear Regression with L1 norm	0.918	1.003
		Linear Regression with L2 norm	0.860	1.023
	1/f Exponent	Linear Regression with L1 norm	0.865	1.048
		Linear Regression with L2 norm	0.822	1.064
	DFA Exponent	Linear Regression with L1 norm	0.826	1.009
		Linear Regression with L2 norm	0.786	1.050
	fEI	Linear Regression with L1 norm	0.731	**0.960**
		Linear Regression with L2 norm	0.683	0.988
	Coherence	Linear Regression with L1 norm	0.800	1.027
		Linear Regression with L2 norm	0.755	1.042
	Imaginary Coherence	Linear Regression with L1 norm	0.740	1.055
		Linear Regression with L2 norm	0.694	1.042
	Phase Locking Value	Linear Regression with L1 norm	0.791	1.025
		Linear Regression with L2 norm	0.741	1.018
	Weighted Phase Lag Index	Linear Regression with L1 norm	0.878	1.045
		Linear Regression with L2 norm	0.821	1.072
	Power Envelope Correlation	Linear Regression with L1 norm	0.876	1.082
		Linear Regression with L2 norm	0.815	1.123
Sensory Perception Quotient (Vision)	All Features	Linear Regression with L1 norm	0.545	1.125
		Linear Regression with L2 norm	0.396	1.282
	Power	Linear Regression with L1 norm	0.890	1.116
		Linear Regression with L2 norm	0.799	1.104
	Theta/Beta Ratio	Linear Regression with L1 norm	0.999	1.005
		Linear Regression with L2 norm	0.990	1.020
	Asymmetry	Linear Regression with L1 norm	1.000	1.000
		Linear Regression with L2 norm	0.983	1.036
	Peak Alpha Frequency	Linear Regression with L1 norm	0.951	1.028
		Linear Regression with L2 norm	0.869	1.050
	1/f Exponent	Linear Regression with L1 norm	0.970	1.022
		Linear Regression with L2 norm	0.908	1.067
	DFA Exponent	Linear Regression with L1 norm	0.890	1.116
		Linear Regression with L2 norm	0.837	1.167
	fEI	Linear Regression with L1 norm	0.832	1.158
		Linear Regression with L2 norm	0.798	1.202
	Coherence	Linear Regression with L1 norm	0.811	0.995
		Linear Regression with L2 norm	0.778	0.984
	Imaginary Coherence	Linear Regression with L1 norm	0.859	1.028
		Linear Regression with L2 norm	0.842	1.054
	Phase Locking Value	Linear Regression with L1 norm	0.819	1.011
		Linear Regression with L2 norm	0.785	1.004

(Continued on next page)

	Weighted Phase Lag Index	Linear Regression with L1 norm	0.901	1.009
		Linear Regression with L2 norm	0.891	1.005
	Power Envelope Correlation	Linear Regression with L1 norm	0.915	1.053
		Linear Regression with L2 norm	0.888	1.063
Sensory Perception Quotient (Auditory)	All Features	Linear Regression with L1 norm	0.614	1.171
		Linear Regression with L2 norm	0.453	1.332
	Power	Linear Regression with L1 norm	0.906	1.105
		Linear Regression with L2 norm	0.857	1.154
	Theta/Beta Ratio	Linear Regression with L1 norm	1.000	1.000
		Linear Regression with L2 norm	0.999	1.012
	Asymmetry	Linear Regression with L1 norm	0.995	1.005
		Linear Regression with L2 norm	0.967	1.037
	Peak Alpha Frequency	Linear Regression with L1 norm	0.950	1.004
		Linear Regression with L2 norm	0.894	0.994
	1/f Exponent	Linear Regression with L1 norm	0.998	1.008
		Linear Regression with L2 norm	0.932	1.076
	DFA Exponent	Linear Regression with L1 norm	0.857	1.119
		Linear Regression with L2 norm	0.818	1.150
	fEI	Linear Regression with L1 norm	0.839	1.121
		Linear Regression with L2 norm	0.802	1.124
	Coherence	Linear Regression with L1 norm	0.874	1.021
		Linear Regression with L2 norm	0.832	1.030
	Imaginary Coherence	Linear Regression with L1 norm	0.903	1.115
		Linear Regression with L2 norm	0.868	1.157
	Phase Locking Value	Linear Regression with L1 norm	0.867	1.016
		Linear Regression with L2 norm	0.835	1.030
	Weighted Phase Lag Index	Linear Regression with L1 norm	0.905	1.048
		Linear Regression with L2 norm	0.885	1.053
	Power Envelope Correlation	Linear Regression with L1 norm	0.920	1.059
		Linear Regression with L2 norm	0.886	1.090

Errors are mean normalized absolute errors. Numbers in **bold** indicate significantly better performance than predicting the mean (One-sided Wilcox test with FDR correction for the 12 feature types for a given model type)

References

[1] Steven K Sutton and Richard J Davidson. "Prefrontal Brain Asymmetry: A Biological Substrate of the Behavioral Approach and Inhibition Systems". In: *Psychological science* 8.3 (1997), pp. 204–210.

[2] Thomas Donoghue et al. "Parameterizing neural power spectra into periodic and aperiodic components". In: *Nature Neuroscience* 23.12 (2020), pp. 1655–1665.

[3] Richard Hardstone et al. "Detrended fluctuation analysis: A scale-free view on neuronal oscillations". In: *Frontiers in Physiology* (2012), pp. 1–13.

[4] Christopher Schölzel. *Nonlinear measures for dynamical systems (Nolds)*. 2019.

[5] Hilgo Bruining et al. "Measurement of excitation-inhibition ratio in autism spectrum disorder using critical brain dynamics". In: *Scientific Reports* 10.1 (2020), pp. 1–15.

[6] Paul L. Nunez et al. "EEG coherency I: Statistics, reference electrode, volume conduction, Laplacians, cortical imaging, and interpretation at multiple scales". In: *Electroencephalography and Clinical Neurophysiology* 103.5 (1997), pp. 499–515.

[7] Guido Nolte et al. "Identifying true brain interaction from EEG data using the imaginary part of coherency". In: *Clinical Neurophysiology* 115.10 (2004), pp. 2292–2307.

[8] Jean Philippe Lachaux et al. "Measuring phase synchrony in brain signals". In: *Human Brain Mapping* 8.4 (1999), pp. 194–208.

[9] Cornelis J. Stam, Guido Nolte, and Andreas Daffertshofer. "Phase lag index: Assessment of functional connectivity from multi channel EEG and MEG with diminished bias from common sources". In: *Human Brain Mapping* 28.11 (2007), pp. 1178–1193.

[10] Martin Vinck et al. "An improved index of phase-synchronization for electrophysiological data in the presence of volume-conduction, noise and sample-size bias". In: *NeuroImage* 55.4 (2011), pp. 1548–1565.

[11] Martin Hardmeier et al. "Reproducibility of functional connectivity and graph measures based on the phase lag index (PLI) and weighted phase lag index (wPLI) derived from high resolution EEG". In: *PLoS ONE* 9.10 (2014).

[12] Russell T. Toll et al. "An Electroencephalography Connectomic Profile of Posttraumatic Stress Disorder". In: *The American journal of psychiatry* 177.3 (2020), pp. 233–243.

[13] Joerg F. Hipp et al. "Large-scale cortical correlation structure of spontaneous oscillatory activity". In: *Nature Neuroscience* 15.6 (2012), pp. 884–890.
